# Real-time in vivo dose measurement using ruby-based fibre optic dosimetry during internal radiation therapy

**DOI:** 10.1007/s13246-023-01288-7

**Published:** 2023-07-03

**Authors:** S. Birajdar, W. Zhang, A. Santos, K. Hickson, S. Afshar Vahid

**Affiliations:** 1 Laser Physics and Photonic Devices Laboratories, UNISA STEM, The University of South Australia, Adelaide, SA 5095 Australia; 2 Department of Radiation Oncology, Royal Adelaide Hospital, Adelaide, SA 5000 Australia; 3 School of Physical Sciences, The University of Adelaide, Adelaide, SA 5005 Australia; 4 Australian Bragg Centre for Proton Therapy and Research, Adelaide, SA 5000 Australia; 5 Medical Physics & Radiation Protection Group, SA Medical Imaging, Adelaide, SA 5000 Australia; 6 Allied Health & Human Performance, University of South Australia, Adelaide, SA 5001 Australia

**Keywords:** Radioluminescence, Ruby, Fibre optic dosimetry, Selective internal radiation therapy, Stem effect, In vivo dosimetry

## Abstract

In vivo dosimetry (IVD) in a commonly used liver cancer treatment of selective internal radiation therapy (SIRT) has been done based on the post-treatment image-based dosimetry approach. Real-time IVD is necessary to verify the dose delivery and detect errors during the treatment for better patient outcomes. This study aims to develop a fibre optic dosimeter (FOD) for in vivo real-time dose rate measurement during internal beta radiation therapy, e.g., SIRT. A ruby fibre optic probe was prepared and studied the radioluminescence (RL) characteristics, including its major challenge of stem effect arising from Cherenkov radiation and luminescence from the irradiated fibre. The stem signal was suppressed adequately using the stem removal technique of optical filtering, and only 2.3 ± 1.1% stem signal was contributed to the measured RL signal. A linear dose rate response was observed during the exposure of the ruby probe to varying dose rates using a 6 MeV electron beam and a positron-emitting radionuclide fluorine-18. The ruby exhibited a temporally non-constant RL signal, which increased the RL signal by 0.84 ± 0.29 counts/sec^2^ during the irradiation of the maximum dose rate used in this study of 9 Gy/min for 2 min. The ability of ruby FOD to measure the absolute dose rate with sufficient stem effect suppression and the linear RL dose rate response indicates its suitability for real-time IVD during internal beta radiation therapy. Future work will investigate the time-dependent RL characteristic of ruby and validate post-treatment image-based dosimetry using ruby-based FOD.

## Introduction

Selective internal radiation therapy (SIRT) with a beta emitter, yttrium—90 (Y-90), is a commonly used treatment for unresectable primary and metastatic liver cancers [1-4]. In SIRT, radiation dose is delivered by directly injecting Y-90 microspheres to the liver tumours through a catheter positioned in the hepatic artery as liver tumours are fed primarily with the blood supply from the hepatic artery, and normal liver parenchyma is fed primarily from the portal vein [5]. After the administration of Y-90, quantitative images can be obtained by single photon emission computed tomography/computed tomography (SPECT/CT) or positron emission tomography/computed tomography (PET/CT) to check the Y-90 distribution for the estimation of dose delivered to the patient. Y-90 bremsstrahlung SPECT/CT scanning suffers from low image quality and poor quantitative accuracy [6]. The time of flight (TOF) PET/CT can be used with better resolution [7] and improved quantification than SPECT/CT [8]. However, the post-treatment image-based dose measurement methods detect the unexpected variations in the overall delivered dose only after the treatment. The same scenario exists with internal beta radiation therapy for treating pancreatic cancer in which phosphorous-32 (P-32) microparticles are injected directly into the tumour to deliver the dose. SPECT/CT imaging of P-32 bremsstrahlung radiation is performed after the treatment to check the coverage of P-32 microparticles inside the tumour [9]. From the dosimetric perspective, the knowledge of actual dose delivered is essential to improve the understanding of absorbed dose–effect relationships. Accurate dosimetry methods enable to maximise therapeutic efficacy while minimising toxicity. There is a need to have real-time in vivo dosimetry (IVD) in place to measure the delivered dose during the treatment, detect significant errors and ensure that the treatments are carried out as intended. Implementation of real-time IVD during internal beta radiotherapy can be used to validate the post-treatment image-based dosimetry. To our best knowledge, no such a system exists which measures the in vivo real-time dose during SIRT. Fibre optic dosimetry (FOD) is an attractive option available today for real-time in vivo dose rate measurement during radiation therapy [10].

A FOD system consists of a scintillator, which emits light spontaneously after excitation by ionising radiation, known as radioluminescence (RL). The scintillator is coupled to an optical fibre to guide the RL light emitted during irradiation. In general, the intensity of RL signal is regarded as proportional to the dose rate absorbed by a scintillator [11]. The availability in desirable size of FOD and the real-time dose rate measurement feature makes them suitable for IVD during internal radiation therapy. The small size of FOD probe allows them to be placed inside a catheter inserted in the tumour to deliver the Y-90 microspheres in SIRT or through the endoscope used to inject P-32 microparticles in pancreatic cancer treatment.

One main drawback of FOD is the generation of Cherenkov radiation and fibre luminescence in the irradiated optical fibre called the stem effect. The stem effect is mainly due to Cherenkov radiation [12]. The stem effect adds additional signal to the RL signal from the scintillator and therefore it must be removed. Four stem removal techniques have been used: background fibre method [13], optical filtering technique [14], air core fibre method [15] and temporal separation technique which is compatible only with a pulsed source of radiation [16]. Among these methods, the optical filtering method is the easiest and cheapest method suitable for in vivo applications. The optical filtering method is used effectively when the scintillator has an RL emission spectrum in a longer wavelength region where the Cherenkov radiation is less significant as Cherenkov radiation is dominant in the blue to ultraviolet spectrum region [17]. However, the Cherenkov radiation spectrum is continuous, and there is also light emission due to Cherenkov effect at longer wavelengths [18].

Jordan [19] investigated ruby-based FOD for dose measurement in external beam radiotherapy. It was demonstrated that the dose depth profiles obtained with the ruby detector for 4 MV photon beams and 9–12 MeV electrons were in good agreement with the ionisation chamber data. Teichmann [20] further investigated ruby-based FOD with an external beam radiation source. A slight increase in RL signal with an accumulated dose of 2 Gy was observed. This was considered to be due to the admixture of impurities in ruby other than Cr ^3+^as previously suggested by Bessonova [21]. In the studies by Jordan [19] and Teichmann [20], the temporal separation technique was used to suppress the stem effect, but it is not applicable for internal radiation therapy where a time decaying radioactive source is used. Kertzscher and Beddar [22] tested a ruby-based fibre optic detector for IVD during high dose rate (HDR) brachytherapy. Depending on the admixture of impurities in ruby, time-dependent (non-constant) scintillation was observed during 50 Gy irradiation with Iridium-192 (Ir-192) and the stem removal technique of background fibre method was used. In the background fibre method, a second optical fibre without a scintillator, i.e., a background fibre is employed parallel to the ruby detector to measure the stem signal. The background fibre technique is not suitable for in vivo applications as it makes the FOD system bulky. Kertzscher and Beddar [22] concluded by simulations that the stem signal suppression would be better by narrowing the bandpass wavelength region of the bandpass filter to ≤ 20 nm when the optical filtering technique is used. Using a scintillator such as a ruby which has a narrow RL main emission peak at 694 nm with a narrow bandpass filter will effectively suppress the stem effect, making the FOD technique suitable for IVD during internal radiation therapy.

The objective of this study is to assess the potential use of ruby FOD during SIRT for real-time dose measurement to validate the image-based dosimetry. We have investigated the stem effect suppression using the optical filtering stem removal technique with a filter of 10 ± 2 nm bandpass wavelength. The RL characteristics of ruby FOD have been studied with the therapeutic 6 MeV electron beam using a linear accelerator (LINAC) and a readily available positron-emitting radiopharmaceutical fluorodeoxyglucose [F-18] FDG.

## Methods

### Fibre optic dosimetry system

**Fig. 1:**
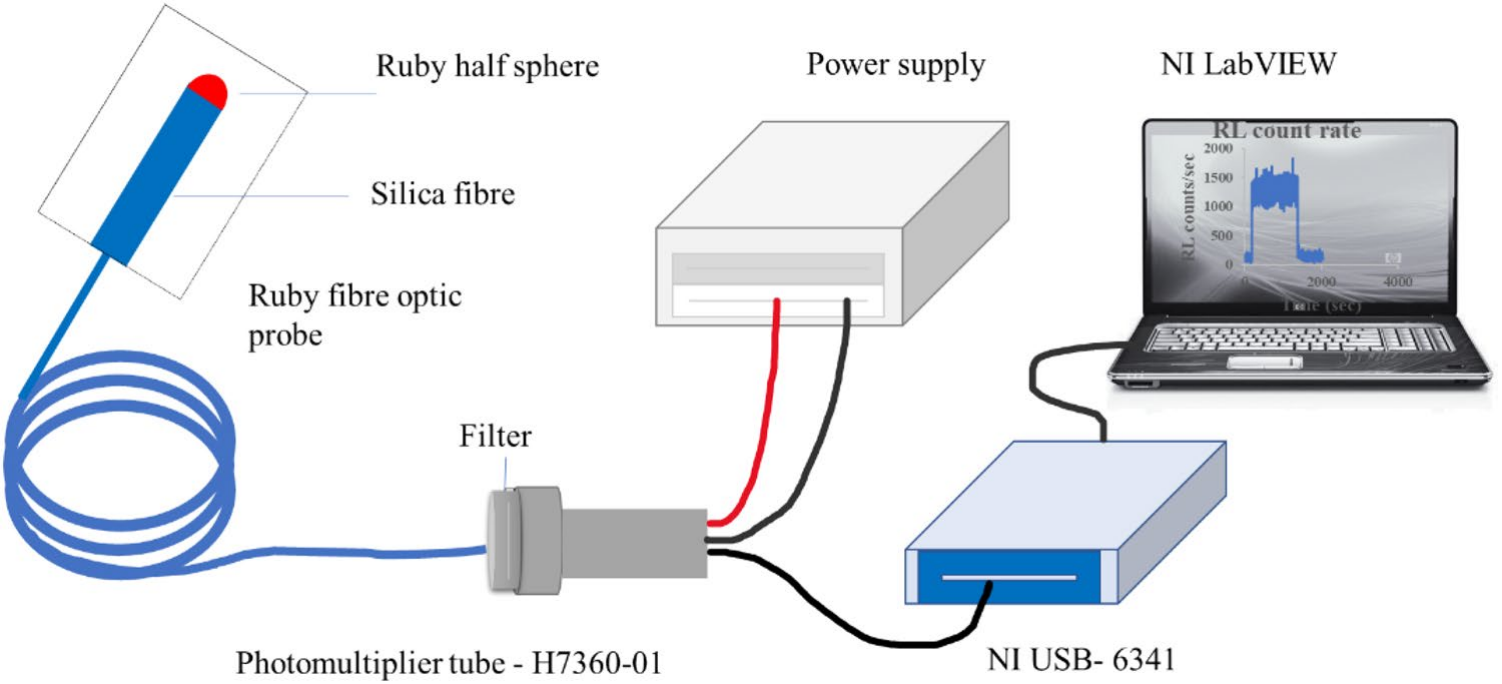
Experimental set-up of the ruby fibre optic dosimetry system

The experimental set-up of the fibre optic dosimetry system is illustrated in Fig. 1. A ruby FOD probe was fabricated by attaching a half-sphere ruby of 1 mm diameter (49,558, Edmund optics Inc, USA) to a 15 m long silica fibre (FP600ERT, Thorlabs) with optical glue (NOA61, UV-curing glue, Thorlabs). The length of the silica fibre was 15 m to transmit the RL signal emitted by ruby to the light detection system or a reader, outside the irradiation facility. The ruby FOD probe is coupled to the reader via a multimode connector (B30670G3, Thorlabs).

The reader consists of a photomultiplier tube (H7360-01, Hamamatsu, Japan) and a data acquisition card (DAQ), National Instruments (USB-6341, National Instruments Inc. USA) with four 32—bit counter and time base of 100 MHz. A customised LabVIEW™ software (National Instruments Inc. USA) reads the counters of USB—DAQ and displays the count rate in proportion to RL light. The sampling rate of 1 Hz, with 1 s integration time, is used throughout the experiments.

The stem effect removal method of optical filtering is used for all the measurements. A bandpass filter FL694.3–10 (Thorlabs Inc, USA), 6.3 mm in thickness, with 694.3 ± 2 nm centre wavelength and 10 ± 2 nm FWHM bandpass, is placed between the end of the fibre and the photomultiplier tube. All measurements are carried out at room temperature and in a dark room to minimise light contamination, which contributes background signal.

### Radioluminescence response using 6 MeV electron beam

**Fig. 2:**
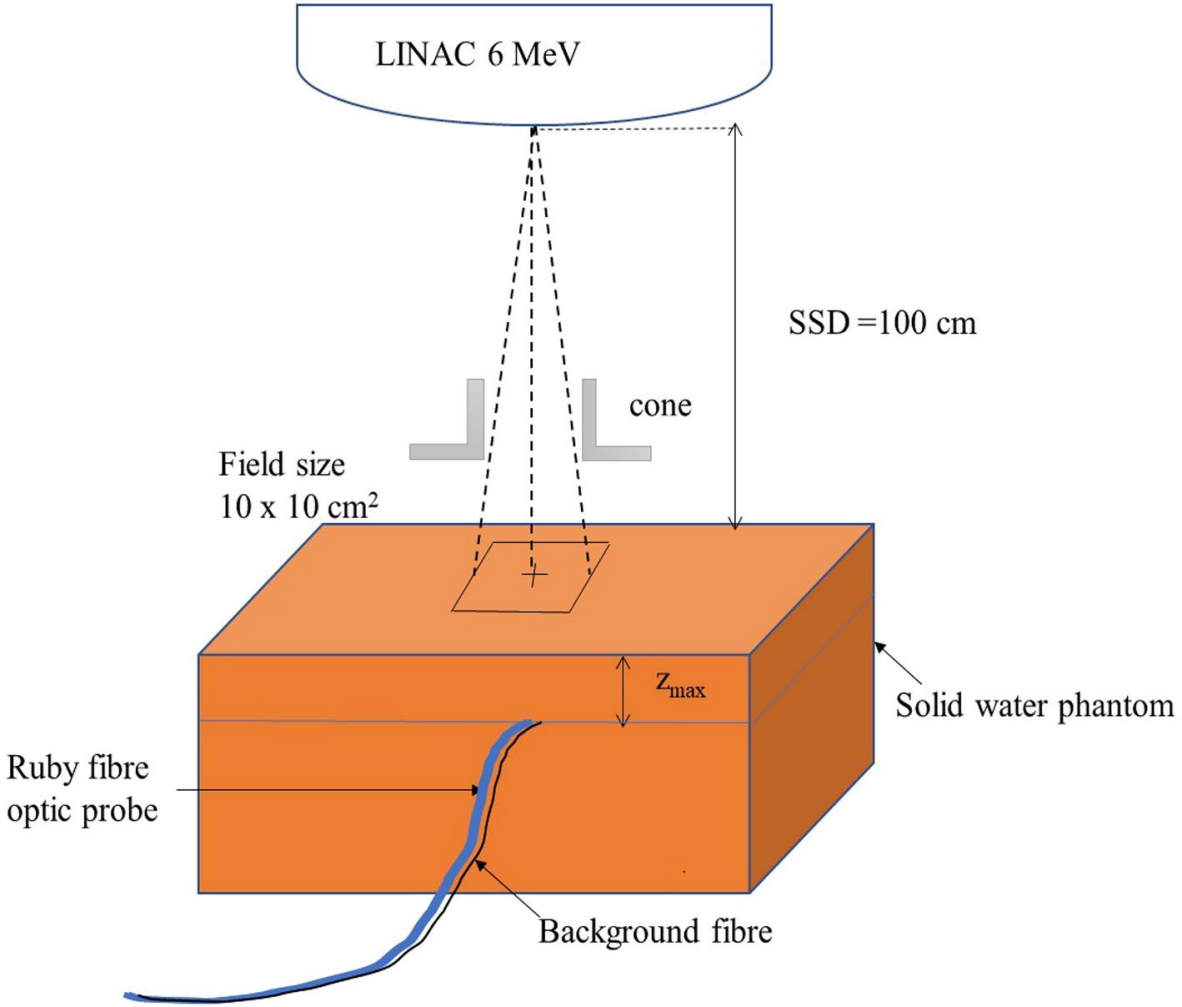
Set-up of the solid water phantom with the ruby FOD and background fibre placed perpendicular to the beam and in the central axis of the 10 × 10 cm^2^ field size for 6 MeV electron beam measurements

A 6 MeV electron beam from a TrueBeam (Varian Medical Systems, Palo Alto, CA) linear accelerator (LINAC) is used to study the stem effect and RL dose rate response of the ruby FOD system. The measurements were performed by placing the ruby FOD probe at a depth of 1.3 cm in a solid water phantom (Gammex RMI, Middleton, U.S.A) at the centre of a field size of 10 × 10 cm^2^ and at a source to surface distance (SSD) of 100 cm as shown in Fig. 2. The electron beam output is specified at the maximum depth dose (*Z*_max_), 1.3 cm for a 6 MeV electron beam in the solid water phantom. The dose rate was varied by altering the repetition rate of the LINAC and the lowest repetition rate is 1 Gy/min at reference conditions. The average energy of a 6 MeV electron beam from the LINAC used in this study is 2.99 MeV. It is related to the *R*_50_, the depth at which the absorbed dose falls to 50% of the maximum dose and *R*_p_, the practical range of a 6 MeV electron beam. The average energy is estimated using *R*_50_ = 2.39 cm and *R*_p_ = 2.81 cm for the 6 MeV electron beam used in this study [23].

#### Stem effect

The background fibre stem removal method with a second silica fibre (FP600ERT, Thorlabs), 15 m long without a scintillator, i.e., a background fibre, is employed to evaluate the stem effect contribution in the filtered ruby RL signal, which is still allowed by the optical filtering stem removal technique. The ruby FOD probe was connected to the reader, and the filtered ruby RL signal using a bandpass filter was measured when irradiated with a 6 MeV electron beam with a dose rate of 9 Gy/min. The background fibre replaced the ruby FOD probe to measure the stem signal with the bandpass filter when irradiated with a 6 MeV electron beam with a dose rate of 9 Gy/min. The light detected by the background fibre approximates the stem signal contribution in the filtered ruby RL signal, which the optical filtering technique could not remove.

#### Dose rate linearity and stability of radioluminescence signal

Linear RL response with changing dose rate is required to effectively use ruby FOD as the RL intensity is proportional to the dose rate. This feature is verified by exposing ruby FOD with varying dose rates, from 1, 3, 5, 7 and 9 Gy/min for 2 min. Also, RL signal stability with accumulated dose has been examined from this same data.

### Radioluminescence response using fluorine-18

**Fig. 3:**
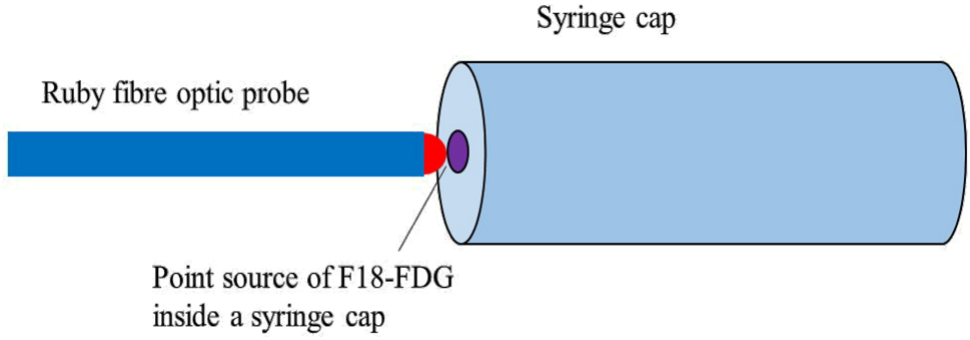
Set up of ruby probe and F18-FDG source

The RL response of the ruby FOD system to a continuously decaying radioisotope was studied with a positron-emitting F-18 source. An unsealed F-18 source with the initial activity of 334 MBq and volume ~ 0.02 ml was placed inside a polypropylene syringe cap to create a point source. The ruby probe was positioned opposite the cap in the immediate vicinity, as shown in Fig. 3. RL count rate response of ruby FOD with the time decaying activity of F-18 is investigated by collecting RL signal data for 3.5 h, i.e., about two half-lives of F-18.

## Results

### Radioluminescence response using 6 MeV electron beam

#### Stem effect

**Fig. 4:**
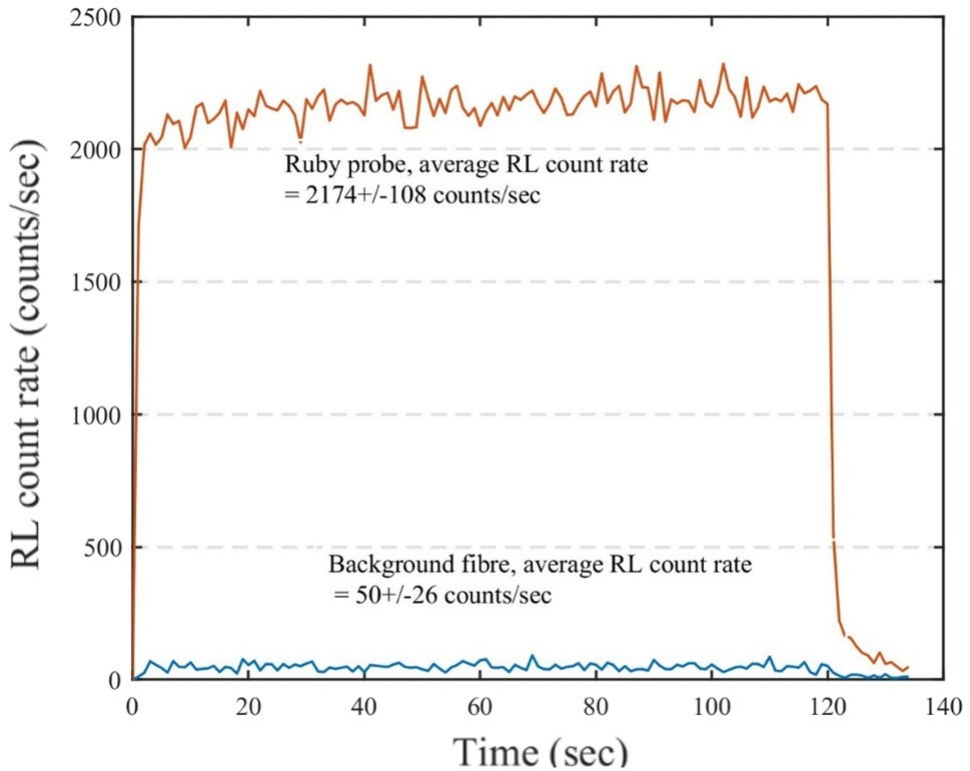
RL count rate from ruby FOD probe and background fibre at 9 Gy/min dose rate

Figure 4 shows the RL signal, i.e., light output in terms of counts/sec or count rate, from the ruby FOD probe and the background fibre when irradiated with a 6 MeV electron beam with a dose rate of 9 Gy/min for 2 min. From the average RL count rate measured by background fibre and ruby FOD probe with uncertainties corresponding to two standard deviations, it is estimated that 2.3 ± 1.1% of the ruby RL signal comes from the stem effect allowed by the optical filtering technique. The afterglow effect, i.e., luminescence after the irradiation, was observed for the ruby.

#### Dose rate linearity and stability of radioluminescence signal

**Fig. 5:**
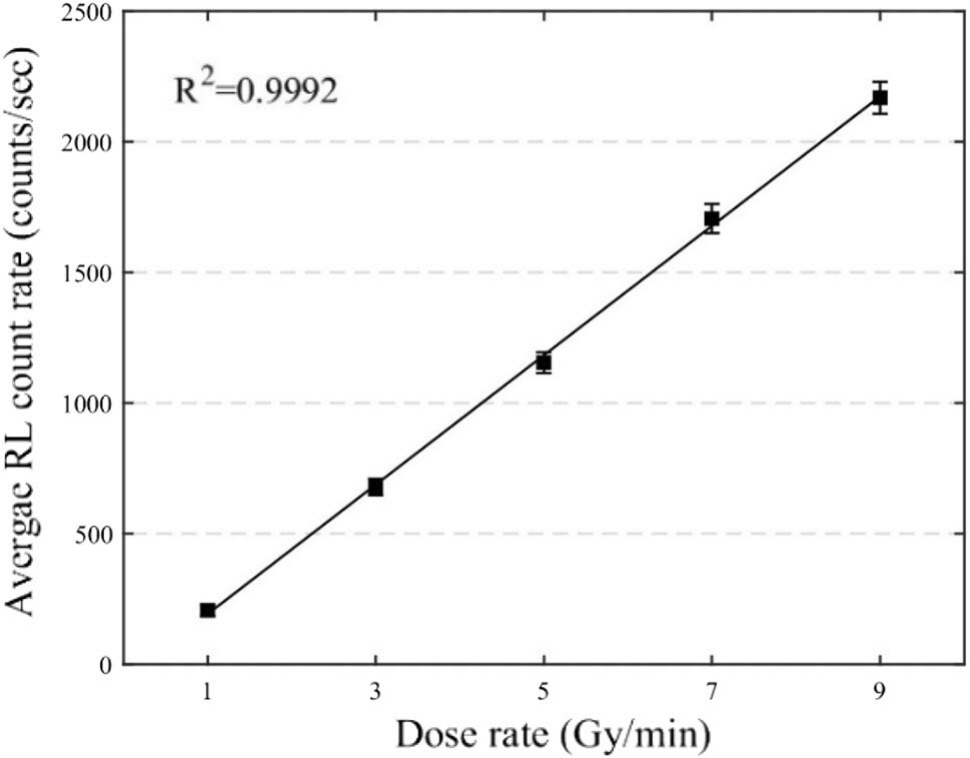
The response of ruby FOD to 1–9 Gy/min irradiation dose dates. The error bars represent  ±  1 standard deviation of RL count rates

The dose rate linearity was assessed from the response of the ruby FOD probe upon irradiation with a 6 MeV electron beam, dose rates of 1, 3, 5, 7 and 9 Gy/min, for two minutes, approximately 1 min apart. Figure 5 plots the average RL count rates against the exposure dose rates. The RL count rate response was observed to be linear over the investigated dose rate interval with *R*^2^ of 0.9992.

**Fig. 6:**
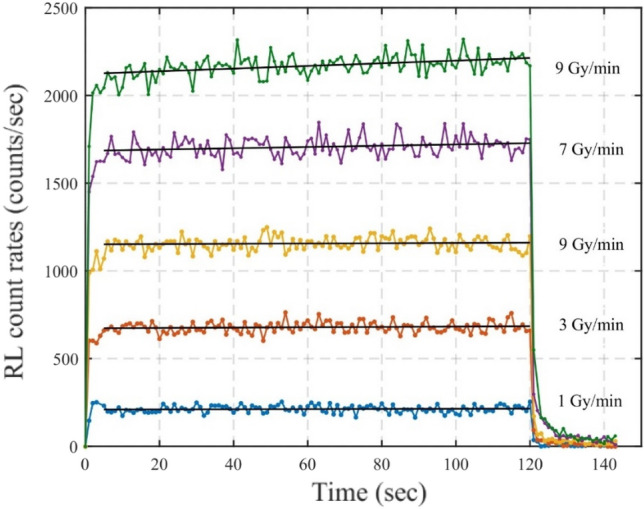
RL count rate response with time for 1 to 9 Gy/min dose rates

The stability of the RL signal with accumulated dose was investigated from the same data used to check the dose rate linearity where the ruby FOD probe was irradiated with 1, 3, 5, 7, and 9 Gy/min dose rate for 2 min using the LINAC 6 MeV electron beam as shown in Fig. 6. The exponential saturation curve is observed just after the irradiation starts, as it takes a while for the RL signal to be stable. The exponential decay curve, i.e., the afterglow effect, is observed when the beam is turned off. Once stable and during the beam-on part, a non-constant linear RL signal was observed, as shown in the beam on part of Fig. 6. A linear regression approach is used during the beam-on part, where RL signal changes linearly with the time to estimate the increase in the count rate per second while the ruby FOD probe is exposed to a constant dose rate. Linear regression estimates the increase in RL count rate per second with uncertainties corresponding to two standard deviations of about 0.040 ± 0.11, 0.12 ± 0.16, 0.18 ± 0.21, 0.41 ± 0.29, and 0.84 ± 0.29 counts/sec^2^ during the irradiation for 2 min with the dose rate of 1, 3, 5, 7, 9 Gy/min, respectively.

The afterglow effect was observed for at least 20 s, as shown in Fig. 6. The average afterglow half-life for ruby scintillator, after the irradiation of dose rate 1, 3, 5, 7 and 9 Gy/min for 2 min, estimated by fitting the exponential decay to the data with uncertainties corresponding to two standard deviations, is found to be 0.57 ± 0.1 s. These results are consistent with the afterglow half-life of the ruby of 3 ms estimated by Jordan [19] and 2.54 ± 0.03 ms and 46.6 ± 0.6 ms by Teichmann [20] using external beam radiotherapy.

### Radioluminescence response using fluorine-18

**Fig. 7:**
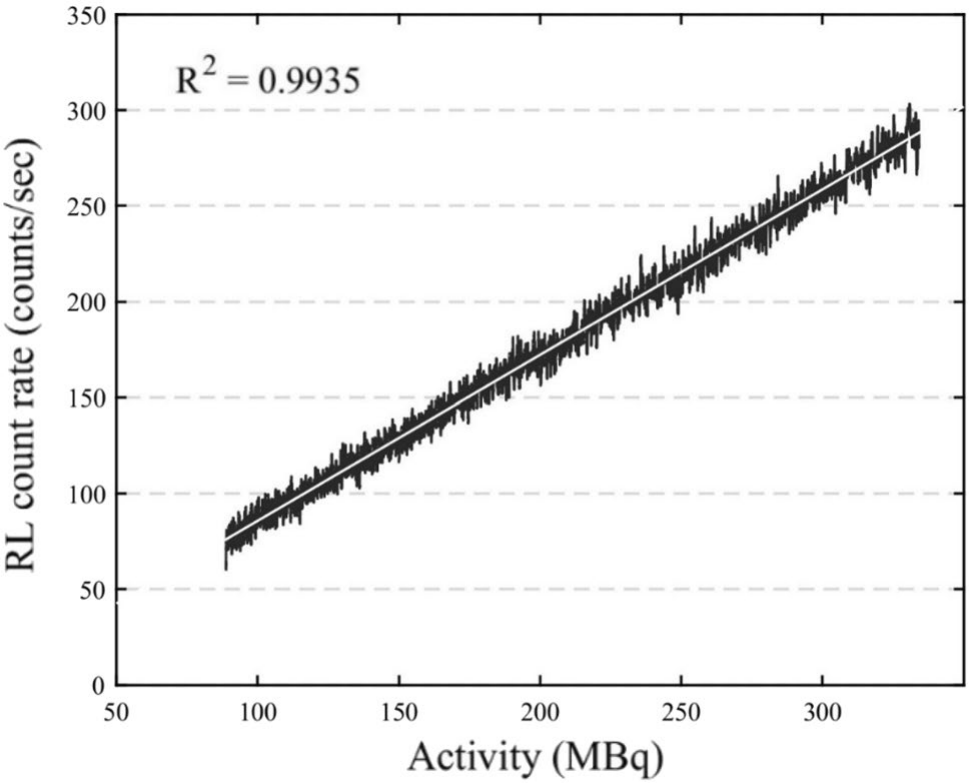
RL count rate response to the activity of F-18, ${A}_{t}$

The initial activity of F-18, $A_{0} ,$ decays with time '*t'* according to the fundamental radioactive decay law. The activity at a time '*t'* $A_{t}$, is specified as, $A_{t} = A_{0} e^{ - \lambda t}$, where $\lambda$ is the decay constant of F-18. F-18 decays to O-18 with the emission of positrons with an average energy of 0.241 MeV per decay [24]. As the F-18 with initial activity, $A_{0} = 334 {\text{MBq}}$ decays with time, the ruby FOD was simultaneously exposed to the varying dose rates in proportion to the activity of F-18 at time '*t'*, ${A}_{t}$. Figure 7 shows the RL response in terms of count rate to the time decaying activity ${A}_{t}$ of F-18. The linear regression analysis approach is used to find the relationship between RL count rate and the time decaying activity ${A}_{t}$ of exposure. *R*^2^ = 0.9935 represents the linear RL response of ruby FOD to the activity ${A}_{t}$ of F-18, ranging from the initial activity of 334 MBq to 89 MBq, after 3.5 h.

From Fig. 7, The RL count rates, ranging from 76 to 288 counts/sec, are obtained when the ruby probe was exposed to 89–334 MBq activity of F-18.

By rearranging the RL count rate data from used to plot Fig. 7, the decay constant for F-18 is evaluated to estimate the half-life of F-18. The measured half-life of F-18 from the RL count rate data obtained during the first two hours with uncertainties corresponding to two standard deviations, 109 ± 1 min agrees with the 109.77 min reported in the literature [25], which indicates the linear response of ruby FOD to the decaying activity of F-18.

## Discussion

This study aimed to assess the use of ruby FOD for the real-time dose rate measurement during internal beta radiation therapy by checking its desirable characteristics. The stem signal, a major disadvantage of FOD is effectively supressed using stem removal technique of optical filtering with a narrow bandpass filter. The stem signal contribution was found to be 2.3 ± 1.1% of the ruby RL signal which is negligible compared to fluctuations in the ruby RL signal associated with the FOD system noise. This finding agrees with previous studies, which have shown by simulations that narrowing the wavelength region of bandpass filter improves the stem signal suppression in ruby-based FOD system [22].

The dose rate linearity has been demonstrated for the investigated range of dose rates, as shown in Fig. 5. Figure 5 shows that the linear fit doesn’t pass through the origin, and it may indicate a threshold dose rate of the ruby dosimeter, which is needed to produce enough light that the dosimeter can detect. Another reason for linear fit passing through the x-intercept could be because of the increased RL sensitivity for the 3, 5, 7, and 9 Gy/min dose rates. Kertzscher and Beddar [22] showed that the RL signal from pre-dosed ruby with 65 Gy after the pause of 500 s was increased by 4%. In this study, the same ruby probe was irradiated with 1 Gy/min followed by 3, 5, 7, and 9 Gy/min with a pause of one minute, which might have contributed to the increased RL sensitivity for the 3, 5, 7, and 9 Gy/min dose rate measurements.

A temporally non-constant RL signal and afterglow effect have been observed as illustrated in Fig. 6. Bessonova [21] suggested that the ruby RL intensity builds up during the constant dose rate exposure depends on the admixture of impurities in the ruby crystal, and the introduction of 0.5% vanadium to the ruby crystal prevents the build-up. Bessonova [21] speculated that the introduction of Ti^3+^ V^3+^ and Mn^3+^ ions prevent the mechanism of causes the RL build-up. The manufacturer has provided that the Ti, Mg and Mn are the main impurities in the ruby scintillator used in this study. The time dependence of the RL signal observed in this study could be that the ruby does not contain the correct admixture of impurities. However, for the lowest dose rate of the 1 Gy/min, the ruby RL signal appears stable.

The non-constant RL and afterglow effect exhibited by the ruby dosimeter could be due to the presence of shallow traps, deep traps, and non-radiative traps in addition to dosimetric traps, which we are interested in for dosimetry. These traps interfere with the luminescence from the dosimetric traps, resulting in a non-stable RL signal and afterglow. Similar characteristics have been observed in carbon-doped aluminium oxide (Al_2_O_3_:C) [26-28]. An exponential saturation curve at the start of irradiation and afterglow after the irradiation is observed for ruby, as shown in Fig. 6, indicating the influence of shallow traps [26]. When the irradiation starts, shallow traps compete with the recombination process and a stable RL signal is not reached before the shallow traps have been filled, which explains the existence of an exponential saturation curve at the start of irradiation. When the irradiation stops, electrons are released from shallow traps, i.e., the emptying process, which gives rise to the phosphorescence signal, i.e., afterglow observed at the end of the irradiation. Kertzscher and Beddar [22] showed that the afterglow effect was a source of significant uncertainty in the dose measurement using a ruby dosimeter during HDR brachytherapy. The effect of afterglow on the dose measurement using a ruby fibre probe during Y-90 internal radiation therapy needs further investigation, which will be explored in future works.

The stability of the RL signal during the irradiation is investigated by fitting a regression line to the data, as shown in Fig. 6, which indicates that the RL signal increases with the accumulated dose. A similar behaviour is observed in Al_2_O_3_:C due to the presence of deep traps which compete with recombination centres. Deep traps can capture and store charge to give rise a light yield that changes with the crystal’s dose history. This effect of the increase in RL signal during prolonged irradiation is called the memory effect [27]. The ruby dosimeter likely contains the deep traps, and RL sensitivity increase with accumulated dose is related to the memory effect. The pre-irradiation technique has been proposed in the case of Al_2_O_3_:C to overcome the sensitivity issue [28]. In this method, Al_2_O_3_:C was pre-dosed with ~  20 Gy before the measurement to saturate all the deep traps and stabilise the concentration of recombination centres, resulting in a stable RL response to a constant dose rate. The pre-irradiation technique could be used in the case of a ruby dosimeter to fill the deeper traps to achieve a more reproducible RL response. Kertzscher and Beddar [22] investigated the pre-irradiation technique to remove the time dependence of the RL signal for the ruby dosimeter. For that, ruby was pre-irradiated with 65 Gy before irradiating for the second time with a pause of 500 s with the same dose. The pre-irradiated ruby maintained the strong time-dependent scintillation and the RL signal was ~ 4% greater than during the first irradiation. Kertzscher and Beddar did not recommend the pre-irradiation to remove the time dependence of the RL signal. Outside of these conditions, pre-irradiation may be helpful for ruby at specific combinations of doses. More investigation is needed, however, if pre-irradiation is adopted for the ruby dosimeter.

Conventionally, post treatment Y-90 image-based dosimetry used in SIRT performed using Medical Internal Radiation Dose (MIRD) schema. According to MIRD, the dose rate absorbed by the tissue upon exposure to radionuclide varies directly with the activity in the tissue [29], and the mean absorbed dose to tissue over the dose integration period is calculated from the time-integrated activity of radionuclide [30]. Recently developed more accurate voxel-based dosimetry with dose point kernel (DPK) is the widely used method for image-based dosimetric calculations, which uses the activity quantification based on tomographic imaging [31]. The image-based dosimetry relies on the amount of activity for the estimation of absorbed dose rate. The linear RL response to the activity is the desirable characteristic required to the validate of image-based dosimetry using FOD. The results in Fig. 7 confirms that the ruby RL response is linear to the investigated range of activity of F-18. The absorbed dose rate also depends on the average energy of emitted particle per decay. The average energy of beta emission from Y-90 is 0.932 MeV per decay [24]. Figures 7 and 5 demonstrated the linear RL response for the dose rates due to the positron emission from F-18 of average energy 0.241 MeV/decay and the 6 MeV electron beam of 2.99 MeV average energy.

In addition to a linear dose rate response, the ability of the ruby FOD to determine the absolute dose rate with adequate stem signal elimination shows the feasibility of in vivo real-time dosimetry during internal beta radiation therapy. The limitation of ruby FOD was the time-dependence of the RL signal. The strategies to overcome the challenge of non-constant RL signal and afterglow would be to use the ruby sample with the right admixture of impurities responsible for the time-independent RL signal as suggested by Bessonova [21] and explore more on the techniques to overcome time-dependence of RL sensitivity such as the pre-irradiation technique used in the case of Al_2_O_3_:C [29]. Further studies are planned to investigate the desirable characteristics of the ruby FOD system including temperature dependence and RL signal stability by adopting the proposed strategy to validate the image-based dosimetry system used in internal beta radiation therapy.

## Conclusion

A ruby fibre optic probe has been prepared by considering the internal beta radiation treatment procedures and their requirements. It has been found that the ruby FOD has the potential to be used for in vivo real-time dose rate measurement. The most suitable stem removal technique for internal beta radiotherapy, optical filtering, achieves adequate stem signal suppression due to the ruby’s narrow peak RL emission at 693.2 nm. The ruby FOD response was linear over the investigated range of dose rates using an external electron beam of 6 MeV and the activity of F-18. Ruby exhibited the time dependent RL response. More investigation is required regarding impurities in chosen ruby samples responsible for the stable RL response and the technique to remove the time-dependence of RL signal.
